# Host-mediated beneficial effects of phytochemicals for prevention of avian coccidiosis

**DOI:** 10.3389/fimmu.2023.1145367

**Published:** 2023-06-02

**Authors:** Inkyung Park, Hyoyoun Nam, Samiru S. Wickramasuriya, Youngsub Lee, Emma H. Wall, Sripathy Ravichandran, Hyun S. Lillehoj

**Affiliations:** ^1^ Animal Bioscience and Biotechnology Laboratory, United States Department of Agriculture, Beltsville Agricultural Research Center, Beltsville, MD, United States; ^2^ AVT Natural North America, Santa Clara, CA, United States

**Keywords:** broiler chicken, coccidiosis, *Eimeria maxima*, growth, gut health, intestinal immunity, phytochemicals, alternatives to antibiotics

## Abstract

Both *in vitro* and *in vivo* studies were conducted to evaluate the beneficial effects of green tea extract (GT), cinnamon oil (CO), and pomegranate extract (PO) on avian coccidiosis. In experiment (EXP) 1, an *in vitro* culture system was used to investigate the individual effects of GT, CO, and PO on the proinflammatory cytokine response and integrity of tight junction (TJ) in chicken intestinal epithelial cells (IEC), on the differentiation of quail muscle cells and primary chicken embryonic muscle cells, and anticoccidial and antibacterial activities against *Eimeria tenella* sporozoites and *Clostridium perfringens* bacteria, respectively. In EXP 2 and 3, *in vivo* trials were carried out to study the dose-dependent effect of blended phytochemicals (GT, CO, PO) on coccidiosis in broiler chickens infected with *E. maxima*. For EXP 2, one hundred male broiler chickens (0-day-old) were allocated into the following five treatment groups: Control group for non-infected chickens (NC), Basal diet group for *E. maxima-*infected chickens (PC), PC group supplemented with phytochemicals at 50 (Phy 50), 100 (Phy 100), and 200 (Phy 200) mg/kg feed diets for *E. maxima-*infected chickens. For EXP 3, one hundred twenty male broiler chickens (0-day-old) were allocated into the following six treatment groups: NC, PC, PC supplemented with phytochemicals at 10 (Phy 10), 20 (Phy 20), 30 (Phy 30), and 100 (Phy 100) mg/kg feed for *E. maxima-*infected chickens. Body weights (BW) were measured on days 0, 7, 14, 20, and 22, and jejunum samples were used to measure cytokine, TJ protein, and antioxidant enzyme responses at 8 days post-infection (dpi). Fecal samples for oocyst enumeration were collected from 6 to 8 dpi. *In vitro*, CO and PO reduced LPS-induced IL-1β and IL-8 in IEC, respectively, and GT enhanced the gene expression of occludin in IEC. PO at 1.0 and 5.0 mg/mL exerted antimicrobial effect against *E. tenella* sporozoites and *C. perfringens* bacteria, respectively. *In vivo*, chickens fed a diet supplemented with phytochemicals showed enhanced BW, reduced oocyst shedding, and decreased proinflammatory cytokines following *E. maxima* challenge. In conclusion, the combination of GT, CO, and PO in the diet of broiler chickens infected with *E. maxima* induced enhanced host disease resistance including innate immunity and gut health, which contributed to improved growth and reduced disease responses. These findings provide scientific support for the development of a novel phytogenic feed additive formula that enhances the growth and intestinal health of broiler chickens infected with coccidiosis.

## 1 Introduction

With the increasing governmental restriction of growth-promoting antibiotics in commercial poultry production (1, 2), there is a timely need to develop research-based novel feed additives to enhance gut health and mitigate negative disease impacts to increase animal resilience in agriculture. Today, there are wide ranges of nutraceuticals available in the feed industry for agriculture animal production including acidifiers, postbiotics, prebiotics, probiotics, phytochemicals, enzymes, minerals, etc (2, 3). For avian coccidiosis, which is caused by several distinct species of *Eimeria* protozoan parasites with an estimated global economic cost of $ 14 billion (4) there has been much development on using plant-derived phytochemicals. These phytochemicals aim to mitigate the negative impacts of avian coccidiosis caused by several distinct species of *Eimeria* (5).

Plant-derived phytochemicals have been effectively used as natural bioactive compounds in agricultural animal production to improve animal health and productivity (2, 5). The main bioactive compounds of the phytochemicals also referred to as phytobiotics or phytogenics, are composed of polyphenols. Their composition and concentration are diverse and vary according to the plant, parts of the plant, geographical origin, harvesting season, environmental factors, storage conditions, and processing techniques (5). Dietary supplementation with selected phytochemicals, either as a single compound, or a mixture of phytochemicals, in young poultry, exerted beneficial effects on growth performance, intestinal immune response, intestinal barrier integrity, and altered intestinal microbiome of broiler chickens challenged with *Eimeria* spp (2). Not all phytochemicals show beneficial effects and there is a need to investigate the relationship between dose and health effects for each phytochemical since some showed toxicity at high dose ranges *in vitro* and *in vivo* which indicates the importance of understanding many contributing factors that will affect the efficacy of phytochemical feed additives in agricultural animal production (6).

In our previous study (Unpublished), 22 types of phytochemicals were evaluated with the novel *in vitro* screening assays based on immunity and cell growth-promoting activities of macrophages, intestinal cells, and muscle cells. Among them, green tea (GT), cinnamon oil (CO), and pomegranate extract (PO) showed various beneficial activities without any cell toxicity, so we further evaluated these phytochemicals as novel phytogenic feed additives for their health-promoting effects on broiler infected with *Eimeria* spp. The main objectives of the present study were: [1] *in vitro* evaluation of effects of the phytochemicals on the host innate immune response in chicken macrophages and the barrier integrity in chicken intestinal epithelial cells (IEC), on myogenic differentiation of quail muscle cells (QMC) and growth effect on primary chicken embryonic muscle cells (PMC), and antimicrobial ability against *Eimeria maxima* and *Clostridium perfringens*, and [2] the *in vivo* effects of dietary supplementation with the selected phytochemical mixture on growth, intestinal immunity, epithelial integrity and antioxidant enzymes in young broiler chickens infected with *E. maxima*.

## 2 Materials and methods

All animal care procedures were approved by the Beltsville Agricultural Research Center Institutional Animal Care and Use Committee (# 20-014). **Supplementary Figure 1** depicts the schematic outline of the experimental design for these studies. All the phytochemicals utilized in this study were produced and provided by collaborating scientists from the AVT Natural. GT, CO, and PO were dissolved in water, sunflower oil, and DMSO, respectively, for *in vitro* studies. The control group, which received zero concentration of phytochemicals, was treated with only each diluent, serving as a negative control group. For the *in vivo* study, the blend of phytochemicals was formulated in a ratio of 1:1:1 (GT : CO:PO) as granulated form.

### 2.1 Experiment 1

#### 2.1.1 Intestinal epithelial cells culture

IEC (2 × 10^5^/mL, HD11; Micromol) were seeded in 24-well plates and maintained in the Dulbecco’s modified Eagle medium (DMEM)/F-12 (Hyclone, Logan, UT) supplemented with 10% heat-inactivated fetal bovine serum (FBS, Hyclone) and 1% penicillin (10,000 unit/mL)/streptomycin (10 mg/mL, Gibco, Grand Island, NY). The IEC was incubated at 41°C in a humidified atmosphere with 5% CO_2_ and 95% air. After 24 h, lipopolysaccharide (LPS; Sigma-Aldrich Inc, St. Louis, MO) at a concentration of 1.0 µg/mL and each phytochemical (CO, GT, and PO) at concentrations of 0.0, 0.5, 1.0, and 10.0 µg/mL were administrated to each well in the 24-well plates. After incubation for 18 h, lysis buffer (Qiagen, Germantown, MD) and 2-mercaptoethanol (Sigma-Aldrich) were used to harvest all cells. Total RNA was isolated from the IEC using the RNeasy Isolation Kit (Qiagen) in a QIAcube (Qiagen) for performing quantitative real-time polymerase chain reaction (qRT-PCR) analysis. All experiments were replicated independently six times.

#### 2.1.2 Quail muscle cell culture

QMCs (2 × 10^5^/mL) were seeded in 24-well plates as per methods described previously (7). The QMCs were maintained in Medium 199 (Hyclone) containing 10% FBS and 1% penicillin/streptomycin until cells reached 70% confluence. Media in 12 wells were replaced by Medium 199 containing 0.5% FBS with 1% penicillin/streptomycin to induce cell differentiation, and in the remaining 12 wells of the same plate, media were replaced by a basic Medium 199 containing 10% FBS to maintain cell proliferation. Each phytochemical (CO, GT, and PO) at concentrations of 0.0, 0.5, 1.0, and 10.0 µg/mL was administrated to each well in the 24-well plates. After incubation at 41°C in a humidified atmosphere with 5% CO_2_ for 18 h, all cells were collected in lysis buffer and 2-mercaptoethanol. RNA from QMC was isolated using the RNeasy Isolation Kit in the QIAcube for performing qRT-PCR analysis. All experiments were replicated more than six times independently.

#### 2.1.3 Primary chicken embryonic muscle cell culture

Fertilized eggs for the embryonic muscle cell culture were obtained from Moyer’s hatchery (Quakertown, PA). The PMC culture was modified based on the method described by (8). In brief, the eggs were incubated using an automated incubator (GQF 1500 professional, Savannah, GA) set at a temperature of 41°C and a humidity level of 70%. The pectoralis major region of the embryos was extracted at 13 days; it was minced and digested with 0.05% trypsin-EDTA (Sigma-Aldrich) at 37°C for 20 min. The PMCs were washed 2–3 times with Hanks balanced salt solution (Sigma-Aldrich) and seeded (2 × 10^5^/mL) in 24-well plates. The PMCs were maintained in DMEM (Hyclone) containing 10% FBS and 1% penicillin/streptomycin until visual confirmation of 70% confluence was attained. Culture media in 12 wells were replaced as DMEM containing 2% FBS with 1% penicillin/streptomycin to induce cell differentiation, and in the remaining 12 wells of the same plate, media were replaced by basic DMEM containing 10% FBS to maintain cell proliferation. Each phytochemical (CO, GT, and PO) at concentrations of 0.0, 0.5, 1.0, and 10.0 µg/mL was added to each well in the 24-well plates. After incubation at 41°C in a humidified atmosphere with 5% CO_2_ for 18 h, all cells were collected in lysis buffer and 2-mercaptoethanol. Total RNA from the PMC was extracted using the RNeasy Isolation Kit in the QIAcube or performing qRT-PCR analysis. RNA was eluted in 30 μL RNase-free water. All experiments were replicated independently more than six times.

#### 2.1.4 cDNA synthesis

The quantity of RNA was evaluated using the NanoDrop spectrophotometer (Thermo Scientific, Waltham, MA) according to the absorbance at 260 nm wavelength. RNA purity was accepted based on the OD260/OD280 ratio of 1.8 and 2.0. 1 ug of total RNA was then reverse-transcribed to cDNA using the QuantiTect reverse transcription kit (Qiagen). The cDNA samples were aliquoted into cryotubes and were stored at - 20°C.

#### 2.1.5 Analysis of cytokines, tight junction proteins, and markers of muscle cell growth by qRT-PCR

The stored cDNA samples were used to measure the gene expression of cytokines (IL-1β and IL-8) and tight junction (TJ) proteins (occluding and ZO-1) on IEC and growth markers (Pax7 and MyoG) on QMCs and PMCs. qRT-PCR was performed using Applied Biosystems QuantStudio 3 Real-Time PCR Systems (Life Technologies, Carlsbad, CA) and SYBR Green qPCR Master Mix (PowerTrack, Applied Biosystems, Foster City, CA). Oligonucleotide primer sequences and product size used for qRT-PCR are listed in **Table 1**. A melting curve was obtained at the end of each run to verify the presence of a single amplification product without primer dimers. Standard curves were generated using serial, 5-fold dilutions of cDNA. The fold changes in each transcript were normalized to glyceraldehyde-3-phosphate dehydrogenase and are relative to the transcript expression in the unstimulated control group (normalized to 1) using the comparative ΔΔ Ct method as previously described (9).

**Table 1:** Oligonucleotide primer sequences for qRT-PCR.

Type	Target gene	Primer sequence (5´-3´)	PCR product size (kb)
Reference	GAPDH	F-GGTGGTGCTAAGCGTGTTAT	264
		R-ACCTCTGCCATCTCTCCACA	
Proinflammatory	IL-1β	F-TGGGCATCAAGGGCTACA	244
		R-TCGGGTTGGTTGGTGATG	
	IL-8	F-GGCTTGCTAGGGGAAATGA	200
		R-AGCTGACTCTGACTAGGAAACTGT	
	TNFSF15	F-CCTGAGTATTCCAGCAACGCA	292
		R-ATCCACCAGCTTGATGTCACTAAC	
	IFN-γ	F-AGCTGACGGTGGACCTATTATT	259
		R-GGCTTTGCGCTGGATTC	
TJ proteins	Claudin-1	F-CCTGATCACCCTCTTGGGAG	145
		R-GCTGCACTCACTCATTGGCT	
	JAM-2	F-AGCCTCAAATGGGATTGGATT	59
		R-CATCAACTTGCATTCGCTTCA	
	Occludin	F-GAGCCCAGACTACCAAAGCAA	68
		R-GCTTGATGTGGAAGAGCTTGTTG	
	ZO-1	F-CCGCAGTCGTTCACGATCT	63
		R-GGAGAATGTCTGGAATGGTCTGA	
Antioxidant enzymes	CAT	F-ACTGCAAGGCGAAAGTGTTT	222
		R-GGCTATGGATGAAGGATGGA	
	HMOX-1	F-CTGGAGAAGGGTTGGCTTTCT	166
		R-GAAGCTCTGCCTTTGGCTGTA	
	SOD-1	F-ATTACCGGCTTGTCTGATGG	173
		R-CCTCCCTTTGCAGTCACATT	

IL, interleukin; TNFSF, tumor necrosis factor superfamily; IFN, interferon; TJ, tight junction; JAM, junctional adhesion molecule; ZO, zonula occludens. CAT, catalase; HMOX, heme oxygenase; SOD, superoxide dismutase.

#### 2.1.6 Anticoccidial assay against *E. tenella*

The anticoccidial effect of GT, CO, and PO on the sporozoite of *E. tenella* was tested using the sporozoite killing assay as described by (10). Briefly, sporulated oocysts of *E. tenella* were prepared and the oocyst shells were disrupted with 0.5-mm glass beads for 10 s using the Mini-Beadbeater (BioSpec Products, Bartlesville, OK). The released sporocysts were washed in chilled Hanks’ balanced salt solution (Hyclone, Logan, UT) and treated with excystation media (0.25% trypsin and 0.014 M taurocholic acid, pH 7.4) for 1 h to induce the release of sporozoites. The sporozoites (2.5 × 10^5^) in each well of 96-well plate were treated with three different novel phytochemicals (GT, CO, and PO). Two different phytochemical doses (0.1 and 1.0 mg/mL) were tested against freshly prepared live sporozoites and incubated at 41°C for 3 hr. AO/PI staining solution (Nexcelom Bioscience LLC, Lawrence, MA) was then added to each mixture at a 1:1 ratio and live sporozoites were counted with a cell counting chamber (Cellometer, Nexcelom Bioscience LLC).

#### 2.1.7 Antibacterial assay against *C. perfringens*

*Clostridium perfringens* (1 × 10^9^ CFU/mL) were treated with four phytochemicals in brain-heart infusion broth (BD Difco, Sparks, MD). Three concentrations of the phytochemicals (0.5, 1.0, and 5.0 mg/mL) were added to the broth and it was incubated at 41°C for 18 hr under anaerobic conditions. After incubation, 100 µL of the cell suspension was spread on differential reinforced Clostridial agar (BD Difco) plates and the plates were incubated at 41°C for 18 hr under anaerobic conditions. Colonies of growing bacteria on the plates were counted to determine the survival ratios (%) of *C. perfringens* within each treatment group.

### 2.2 Experiment 2

#### 2.2.1 Chickens and experimental design

A total of one hundred newly hatched male broiler chickens (Ross 708) at zero-day old (Longenecker’s hatchery, Elizabethtown, PA) were weighed and allocated to five dietary treatments in a randomized complete block design. Each treatment group had five cages with four chickens per cage (0.65 × 0.75 m^2^). The dietary treatments (**Supplementary Table 1**) included basal diet for non-infected chickens (NC), basal diet for *E. maxima* infected chickens (PC), PC supplemented with a blended phytochemical mixture (Phy) at 50 (Phy 50), 100 (Phy 100), and 200 (Phy 200) mg/kg feed. Based on experiment (EXP) 1, the Phy was manufactured and provided by AVT natural products. During the experimental period, the chickens were provided with ad libitum access to experimental feed and water. The experimental design used for the study is illustrated in **Supplementary Figure 1**.

#### 2.2.2 Determination of body weight

The body weights (BW) of chickens were measured on days 0, 7, 14, 20, and 22 for calculating average daily gain (ADG). Dead chickens were removed and weighed to perform adjustments for the growth data.

#### 2.2.3 Oral administration of *E. maxima*

All chickens, except for the NC group, were orally inoculated with *E. maxima* (10,000 sporulated oocysts/chicken, Beltsville strain 41A) on day 14 as per previously described methods (1). A DNA genotyping test was performed to identify the purity of the infected *E. maxima* (11).

#### 2.2.4 Collection of jejunal samples

One chicken of nearly average BW per each cage was euthanized by cervical dislocation on day 22, and a small section (2 cm) of the distal jejunum without digest was collected and stored in RNAlater^®^ (Invitrogen, Carlsbad, CA) at -20°C until subsequent analysis.

#### 2.2.5 Fecal oocyst shedding

From days 20 to 22 (6 to 8 days post-infection: dpi), fecal samples were collected, and the number of oocysts was counted as per protocols described previously (12) using the McMaster chamber according to the formula below:

Total oocysts/chicken = [counted oocyst × dilution factor × (fecal sample volume/counting chamber volume)]/number of chickens per cage.

#### 2.2.6 Isolation of RNA and reverse transcription from jejunal samples

Total RNA was isolated from the jejunal samples that were stored in the RNAlater^®^ (Invitrogen). Total RNA was extracted using TRIzol reagent (Invitrogen) followed by DNase digestion as described (13). The methods of RNA quantity, RNA purity, and cDNA production in this study were carried out as described previously (14). The cDNA samples were aliquoted into cryotubes and were stored at - 20°C.

#### 2.2.7 Gene expression analysis by qRT-PCR from the extracted RNA

The oligonucleotide primer sequences used for qRT-PCR analysis are shown in **Table 1**. The expression of various cytokines (IL-1β, IL-8, IFN-γ, and TNFSF15), TJ proteins (claudin, JAM-2, occludin, and ZO-1), antioxidant enzymes (CAT, HMOX, and SOD-1) was evaluated in the jejunal samples. The gene expression analysis by qRT-PCR from the extracted RNA was carried out as described previously (15).

### 2.3 Experiment 3

All the methods in EXP 3, except for treatment groups and the number of jejunal samples, were conducted the same as in EXP 2.

#### 2.3.1 Chickens and experimental design

A total of 120 newly hatched male broiler chickens (Ross 708) at zero-day old were used for EXP 3. The chickens were allocated to six dietary treatments in a randomized complete block design. Each treatment group had five cages with four chickens per cage. The dietary treatments included a basal diet without infection (NC), a basal diet with *E. maxima* infection (PC), PC with a blended phytochemical mixture (Phy) at 10 (Phy 10), 20 (Phy 20), 30 (Phy 30), and 100 (Phy 100) mg/kg feed. The experimental design used for the study is illustrated in **Supplementary Figure 1**.

#### 2.3.2 Collection of jejunal samples

Eight chickens of nearly average BW per treatment were euthanized by cervical dislocation on day 22, and a small section (2 cm) of the distal jejunum without digest was collected and stored in RNAlater^®^ (Invitrogen) at -20°C until subsequent analysis.

#### 2.3.3 Statistical analysis

Each response in *in-vitro* was evaluated using the Proc GLM in SAS software version 9.4 (SAS Inc., Cary, NC). Data from the animal trial were analyzed using a mixed model (PROC MIXED) in SAS. Linear or quadratic effects were analyzed through orthogonal polynomial contrasts which were generated using the PROC IML procedure for non-equally spaced doses. NC was excluded in the analysis of the linear and quadratic effects after infection. If a significant value in treatment groups, linear, and quadratic existed, the mean values between treatments were compared in a pairwise manner through the PDIFF option in SAS. The results are reported as least squares mean values ± standard error of the mean. Probability values less than 0.05 were considered significantly different.

## 3 Results

### 3.1 Experiment 1:*in-vitro* study

#### 3.1.1 Proinflammatory cytokine in chicken intestinal epithelial cells

**Figure 1:**
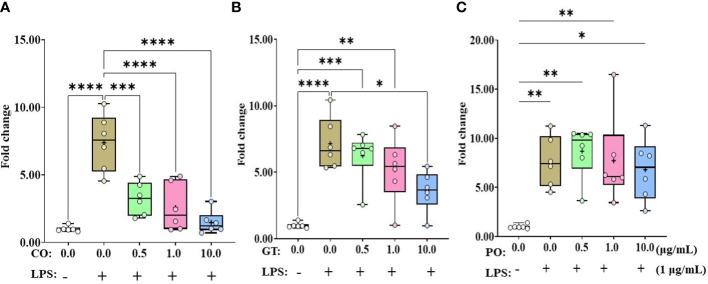
Gene expression of IL-1β in phytochemical-induced chicken intestinal epithelial cells (IECs). The IECs were treated with three different concentrations (0.5, 1.0, and 10.0 mg/mL) of each phytochemical [cinnamon oil (CO, **A**), green tea extract (GT, **B**), and pomegranate (PO, **C**)] for 18 h with LPS (1μg/mL). The data represent the average of six independent experiments. *P* < 0.05 (*), *P* < 0.01 (**), *P* < 0.001 (***), and *P* < 0.0001 (****) were considered statistically significant compared to the control (0.0 mg/mL phytochemicals). The fold changes in each transcript were normalized to glyceraldehyde-3-phosphate dehydrogenase and are relative to the transcript expression in unstimulated control group (normalized to 1) using the comparative ΔΔ Ct method.

Administration of LPS without any phytochemicals increased (*P* < 0.01) IL-1β expression level in IEC more than 7-fold compared to untreated control (**Figure 1**). According to the concentrations of CO administration, the IL-1β expression level was linearly reduced (*P* < 0.001) by the administration of CO with LPS (**Figure 1A**). In the case of GT, only 10.0 µg/mL dose reduced (*P* < 0.05) LPS-induced IL-1β expression level (**Figure 1B**). Administration of PO did not affect IL-1β expression levels compared to that of the LPS group without administration of PO (**Figure 1C**).

**Figure 2:**
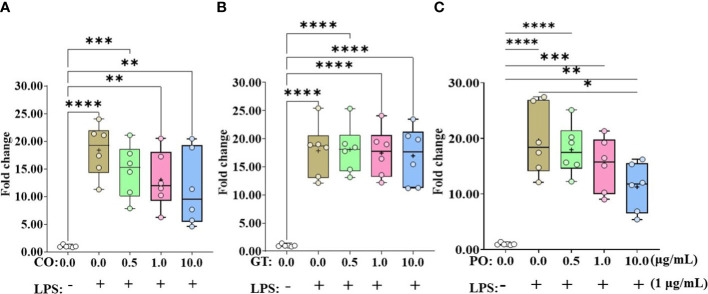
Gene expression of IL-8 in phytochemical-induced chicken intestinal epithelial cells. Cells were treated with three different concentrations (0.5, 1.0, and 10.0 mg/mL) of each phytochemical [cinnamon oil (CO, **A**), green tea extract (GT, **B**), and pomegranate (PO, **C**)] for 18 h with LPS (1μg/mL). The data represent the average of six independent experiments. *P* < 0.05 (*), *P* < 0.01 (**), *P* < 0.001 (***), and *P* < 0.0001 (****) were considered statistically significant compared to the control (0.0 mg/mL phytochemicals). The fold changes in each transcript were normalized to glyceraldehyde-3-phosphate dehydrogenase and are relative to the transcript expression in unstimulated control group (normalized to 1) using the comparative ΔΔ Ct method.

IL-8 expression levels in IEC were elevated by more than 17-fold when administrating LPS without any phytochemicals (**Figure 2**). Administration of PO at 10 µg/mL suppressed (*P* < 0.05) the LPS-induced IL-8 expression level (**Figure 2C**). However, IL-8 expression levels in LPS groups were not affected (*P* > 0.05) by CO and GT treatment (**Figures 2A, B**).

#### 3.1.2 Tight junction proteins in chicken intestinal epithelial cells

Occludin expression level was (*P* < 0.005) increased by 10 µg GT treatment compared to the untreated group (**Table 2**) while administration of CO and PO did not change the occludin expression level. ZO-1 expression level was not changed by the administration of CO, GT, and PO.

**Table 2:** Alteration of mucin and tight junction proteins in chicken intestinal epithelial cells by phytochemicals.

	Concentration of phytochemical, µg/mL				SEM	*P*-value
	0	0.5	1	10		
Occludin, fold change						
CO	1.00	1.06	1.02	1.15	0.24	0.925
GT	1.00^c^	1.17^bc^	1.77^ab^	1.97^a^	0.23	0.001
PO	1.00	1.03	1.02	1.30	0.20	0.393
ZO-1, fold change						
CO	1.00	1.21	1.46	1.95	0.37	0.097
GT	1.00	1.06	1.08	1.04	0.24	0.990
PO	1.00	0.98	0.98	1.00	0.25	0.999

Three different concentrations (0.5, 1.0, and 10.0 mg/mL) of each phytochemical [cinnamon oil (CO), green tea extract (GT), and pomegranate (PO)] were treated into chicken intestinal epithelial cells for 18. The data represent the average of six independent experiments. The fold changes in each transcript were normalized to glyceraldehyde-3-phosphate dehydrogenase and are relative to the transcript expression in unstimulated control group (normalized to 1) using the comparative ΔΔCt method.

#### 3.1.3 Proliferation and differentiation of quail muscle cells and primary chicken embryonic muscle cells

In QMC culture (**Table 3**), reducing the concentration of 10% FBS up to 0.5% showed (*P* < 0.05) higher Pax7 expression levels by more than 2-fold regardless of phytochemical administration compared to that of 10% FBS. 0.5% FBS showed (*P* < 0.05) greater MyoG expression levels compared to that of 10% FBS. When 0.5% FBS was used, treatment with 10 µg/ml PO increased the MyoG expression level from 5 to 8-fold (*P* < 0.05). Other phytochemicals such as CO, and GT did not affect MyoG expression levels. In PMC culture (**Table 4**), unlike QMC, most Pax7 and MyoG expression levels were not changed in the presence of 2% FBS and phytochemical administration. However, treatment with 10 µg GT increased (*P* < 0.05) Pax7 expression level compared to that of 10% FBS.

**Table 3:** Proliferation and differentiation of quail muscle cells by fetal bovine serum concentration and phytochemicals.

	Concentration of FBS, %					SEM	*P*-value
	10	0.5	0.5	0.5	0.5		
	Concentration of phytochemical, µg/mL						
	0	0	0.5	1	10		
Pax7 in QMC, fold change							
CO	1.00^b^	2.46^a^	2.57^a^	2.56^a^	2.71^a^	0.43	0.003
GT	1.00^b^	5.46^a^	5.61^a^	5.61^a^	5.58^a^	0.70	<0.001
PO	1.00^b^	2.22^a^	2.35^a^	2.28^a^	2.46^a^	0.42	0.013
MyoG in QMC, fold change							
CO	1.00^b^	5.87^a^	5.96^a^	5.86^a^	6.18^a^	0.92	<0.001
GT	1.00^b^	5.85^a^	5.83^a^	5.89^a^	5.88^a^	0.67	<0.001
PO	1.00^c^	5.92^b^	6.16^ab^	7.74^ab^	8.62^a^	0.91	<0.001

Three different concentrations (0.5, 1.0, and 10.0 mg/mL) of each phytochemical [cinnamon oil (CO), green tea extract (GT), and pomegranate (PO)] were treated into quail muscle cells for 18 h. The data represent the average of six independent experiments. ^a, b^Means in the same row with different superscripts differ (P< 0.05), and the difference was re-evaluated by PDIFF option in SAS when P-value between treatments was less than 0.05. The fold changes in each transcript were normalized to glyceraldehyde-3-phosphate dehydrogenase and are relative to the transcript expression in unstimulated control group (normalized to 1) using the comparative ΔΔCt method. FBS, fetal bovine serum.

**Table 4:** Proliferation and differentiation of primary chicken embryonic muscle cells by fetal bovine serum concentration and phytochemicals.

	Concentration of FBS, %					SEM	*P*-value
	10	2.0	2.0	2.0	2.0		
	Concentration of phytochemical, µg/mL						
	0	0	0.5	1	10		
Pax7 in PMC, fold change							
CO	1.00	1.76	1.74	1.78	1.78	0.43	0.319
GT	1.00	1.75	1.72	1.74	1.75	0.45	0.397
PO	1.00	1.74	1.75	1.74	1.78	0.38	0.214
MyoG in PMC, fold change							
CO	1.00	1.79	1.83	1.87	1.90	0.45	0.247
GT	1.00	1.85	1.83	1.89	1.88	0.47	0.288
PO	1.00	1.84	1.87	2.53	2.76	0.64	0.086

Three different concentrations (0.5, 1.0, and 10.0 mg/mL) of each phytochemical [cinnamon oil (CO), green tea extract (GT), and pomegranate (PO)] were treated into primary chicken embryonic muscle cells for 18 h. The data represent the average of six independent experiments. The fold changes in each transcript were normalized to glyceraldehyde-3-phosphate dehydrogenase and are relative to the transcript expression in unstimulated control group (normalized to 1) using the comparative ΔΔCt method. FBS, fetal bovine serum.

#### 3.1.4 Anticoccidial with *C. perfringens*

**Figure 3:**
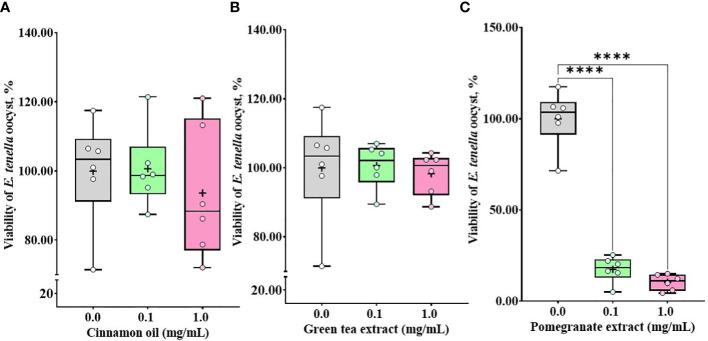
Viability of *E*. *tenella* sporozoites against phytochemical administration. Antiparasitic activities against *E*. *tenella* sporozoites were determined by counting viable sporozoites after 3 h incubation with cinnamon oil **(A)**, green tea extract **(B)**, and pomegranate extract **(C)**. The data represent the average of six independent experiments. *P* < 0.0001 (****) was considered statistically significant compared to the control (0.0 mg/mL phytochemicals). 100% value of the control is 2.23 × 10^7^ ± 2.0 × 10^6^ of *E*. *tenella* sporozoites. Pomegranate administration reduced the viability of sporozoites by less than 20% in the experimental range.

**Figure 4:**
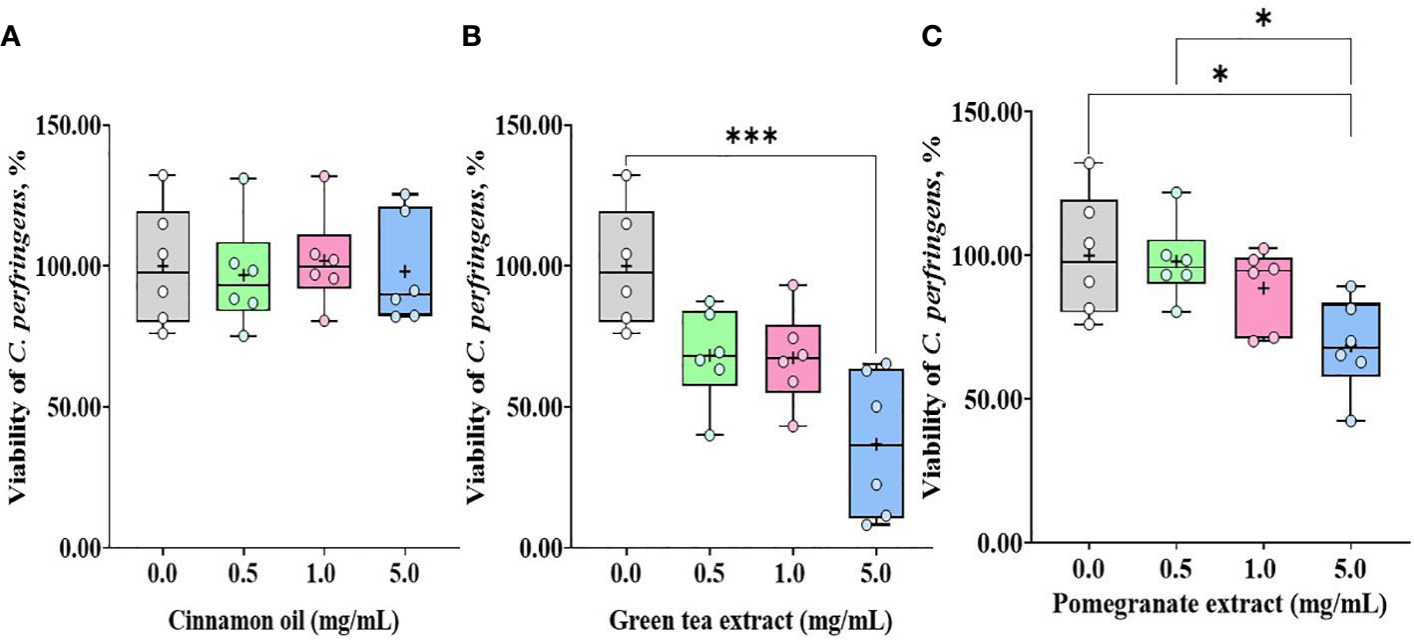
Viability of *C.m perfringens* against phytochemical administration. Antibacterial activities against *C*. *perfringens* were determined by counting colonies after 18 h incubation with cinnamon oil **(A)**, green tea extract **(B)**, and pomegranate extract **(C)**. The data represent the average of six independent experiments. *P* < 0.05 (*) and *P* < 0.001 (***) were considered statistically significant compared to the control (0.0 mg/mL phytochemicals). 100% value of the control is 1 × 10^9^ ± 1.1 × 10^8^ CFU of *C*. *perfringens*. Pomegranate and tea extract at 5.0 mg/mL reduced the viability of *C*. *perfringens* by less than 40%.

Treatment with CO (**Figure 3A**) and GT (**Figure 3B**), regardless of their doses (0.1 and 1.0 mg/mL), did not affect (*P* > 0.05) *E. tenella* sporozoite viability whereas administration of PO (**Figure 3C**) decreased (*P* < 0.0001) the viability of *E. tenella* sporozoites by 83% at 0.1 mg/mL, and 90% at 1.0 mg/mL. *C. perfringens* bacterial counts were not changed (*P* > 0.05) by treating with CO (**Figure 4A**) at three different dose levels (0.5, 1.0, and 5.0 mg/mL). GT at the dose of 5.0 mg/mL decreased (*P* < 0.001) the viability of *C. perfringens* by 63.3% compared to the control group. However, GT at 0.5 and 1.0 mg/mL also numerically decreased (*P* > 0.05) the viability of *C. perfringens* without statistical differences (**Figure 4B**). In PO groups, there were no differences up to 1.0 mg/mL, whereas PO at 5.0 mg/mL decreased the viability of *C. perfringens* compared to 0.0 and 0.5 mg/mL of PO (**Figure 4C**).

### 3.2 Experiment 2: *in vivo* studies

#### 3.2.1 Body weight and average daily gain

Dietary Phy supplementation did not change the BW of chickens until d 7 (**Table 5**). Dietary supplementation with Phy at 200 mg/kg feed decreased (*P* < 0.05) BW on d 14 before infection compared to others. *E. maxima* infection, regardless of phytochemical supplementations, reduced the BW on d 20 and 22 compared to that of the NC group. Phy supplementation at 100 mg/kg feed increased (*P* < 0.05) the BW on d 20 (6 dpi) of chickens infected with *E. maxima* compared to that of the PC group. On d 22 (8 dpi), the dietary Phy supplementation showed significant linear (*P* = 0.002) and quadratic effects (*P* < 0.001) on the final BW. In treatment groups, dietary supplementation with Phy at 50 and 100 mg/kg feed increased the final BW compared to that of the PC group.

**Table 5:** Body weight and average daily gain chickens fed a diet supplemented with a phytochemical mixture (Phy) in experiment 2.

			Phy, mg/kg feed					*P*-value	
	NC	PC	50	100	200	SEM	Trt	Linear	Quadratic
BW, g									
Before infection									
Initial	36.98	36.45	37.02	37.08	36.42	0.29	0.795	0.884	0.295
d 7	155.21	148.77	163.33	157.01	149.28	4.91	0.359	0.377	0.001
d 14	410.98^b^	437.22^ab^	455.34^a^	451.88^a^	406.08^c^	8.83	0.012	0.438	< 0.001
After infection									
d 20 (6 dpi)	723.42^a^	593.33^c^	650.21^bc^	670.78^ab^	606.09^bc^	23.02	0.024	0.819	< 0.001
d 22 (8 dpi)	863.44^a^	674.88^c^	726.34^b^	778.26^b^	632.04^c^	22.64	0.001	0.002	< 0.001
ADG, g									
Before infection									
d 0 to 7	16.91	16.12	18.0	17.13	16.12	0.71	0.397	0.349	0.001
d 7 to 14	36.73	41.03	41.7	42.12	36.56	1.44	0.056	0.497	< 0.001
After infection									
d 14 to 20	51.82^a^	26.20^c^	32.7^bc^	36.71^b^	33.48^bc^	3.01	0.003	< 0.001	< 0.001
d 20 to 22	70.03^a^	40.46^bc^	38.0^bc^	53.00^ab^	13.02^c^	9.12	0.021	< 0.001	< 0.001
d 14 to 22	56.48^a^	29.77^c^	33.9^bc^	40.82^b^	28.41^c^	2.48	< 0.001	0.209	< 0.001

All chickens, except for NC, were infected by oral gavage on day 14 with 1.0 × 10^4^oocysts/chicken of Eimeria maxima. SEM, the standard error of the mean; Phy, a mixture of phytochemicals used in experiment 1, NC, basal diet; PC, basal diet for E. maxima-infected chickens; Trt, treatment; BW, body weight; ADG, average daily gain; d, day; dpi, days post-infection. ^a-c^Means in the same row with different superscripts differ (P < 0.05), and the difference was re-evaluated by PDIFF option in SAS when P-value between treatments was less than 0.05.

Like the BW change according to dietary Phy supplementation, ADG was also affected by the Phy supplementation. Among treatment groups from d 0 to 14, dietary Phy supplementation did not change the ADG of chickens even though the quadratic effect regarding the ADG (*P* < 0.001) significantly appeared in this period. Infection groups with *E. maxima* decreased the ADG of chickens during the whole infection period (d 14 to 22) compared to that of the NC group. During two different periods during *E. maxima* infection which are d 14 to 20 and d 20 to 22, dietary supplementation with the Phy showed (*P* < 0.001) linear and quadratic effects on ADG of chicken dose-dependently. Chickens supplemented with the Phy at 100 mg/kg feed showed (*P* < 0.05) greater ADG between infected groups on d 14 to 20, whereas dietary supplementation with Phy at 200 mg/kg feed lowered the ADG of chickens compared to that of the phytochemical group at 100 mg/kg feed from day 20 to 22. During the entire infection period post-*E. maxima*, from day 14 to 22, dietary supplementation with the Phy induced (*P* < 0.001) quadratic effects on ADG of chicken in a dose-dependent manner, and the mean of ADG was the highest with 100 mg/kg Phy feed, whereas PC and Phy at 200 mg showed the lowest ADG.

#### 3.2.2 Jejunal cytokines, tight junction proteins, and antioxidant enzymes

**Figure 5:**
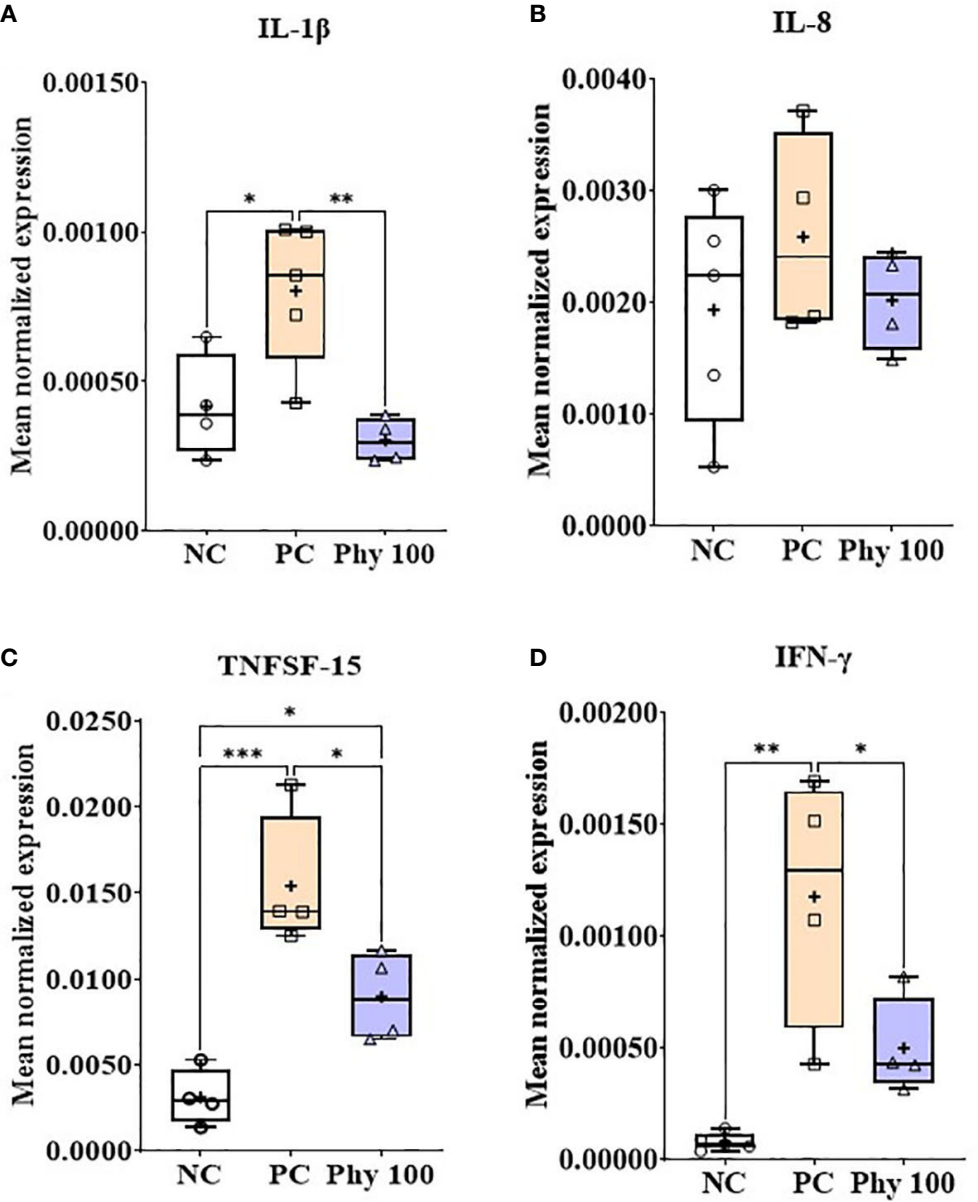
Transcripts of proinflammatory cytokines (**A**; IL-1β, **B**: IL-8, **C**: TNFSF-15 and **D**: IFN-γ) in jejunum of chickens fed diet supplemented with phytochemical mixture at 100 mg/kg feed during infection with *E. maxima* in experiment 2. NC, basal diet; PC, basal diet for infected chickens; Phy 100, phytochemical at 100 mg/kg feed; IL, interleukin; TNFSF, tumor necrosis factor superfamily; IFN, interferon. All chickens, except for NC, were infected by oral gavage on day 14 with 1.0 × 10^4^ oocysts/chicken of *E. maxima*. *P* < 0.05 (*), *P* < 0.01 (**), and *P* < 0.001 (***) were considered statistically significant. The data were collected from jejunal tissues of 5 chickens per treatment on d 22 (8 days post-infection). Transcript levels of the cytokines were measured using quantitative RT-PCR and normalized to GAPDH transcript levels.

**Figure 6:**
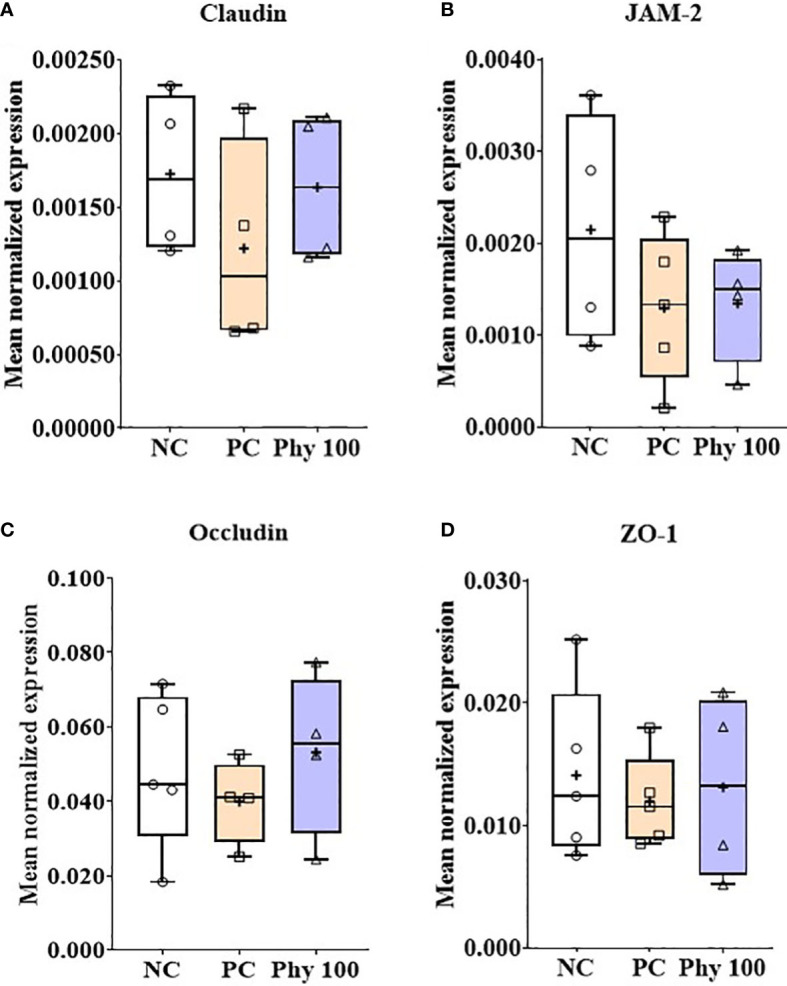
Transcripts of tight junction proteins (**A**; Claudin, **B**: JAM-2, **C**: Occludin and **D**: ZO-1) in jejunum of chickens fed diet supplemented with phytochemical mixture at 100 mg/kg feed during infection with *E. maxima* in experiment 2. NC, basal diet; PC, basal diet for infected chickens; Phy 100, phytochemical at 100 mg/kg feed; JAM, junctional adhesion molecule; ZO, zonula occludins. All chickens, except for NC, were infected by oral gavage on day 14 with 1.0 × 10^4^ oocysts/chicken of *E. maxima*. The data were collected from jejunal tissues of 5 chickens per treatment on d 22 (8 days post-infection). Transcript levels of the cytokines were measured using quantitative RT-PCR and normalized to GAPDH transcript levels.

*E. maxima* infection increased (*P* < 0.05) IL-1β, TNFSF-15, and IFN-γ expression levels in the jejunum of chickens compared to that of NC, while dietary Phy 100 supplementation decreased (*P* < 0.05) these proinflammatory cytokine levels compared to that of PC (**Figure 5**). Jejunal tight junction (TJ) proteins (**Figure 6**) and antioxidant enzymes (**Supplementary Figure 2**) in chickens were not changed by *E. maxima* infection and dietary Phy 100 supplementation.

#### 3.2.3 Oocyst shedding

**Figure 7:**
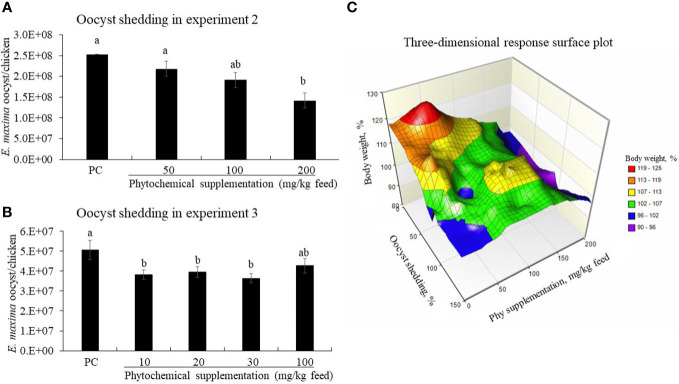
Fecal oocyst shedding **(A, B)** and three-dimensional response plot **(C)** on final body weight (BW) of chicken fed diet supplemented with phytochemical mixture. The oocyst shedding was measured from fecal samples which were collected on a tray installed under the cage from 6 to 8 days post-infection. NC group, basal diet without infection, was excluded in oocyst results because there was no oocyst. In the three-dimensional plot, the average values of PC on final BW and oocyst number were considered as 100%. The surface was drawn with each value of BWs, oocyst numbers, and Phy concentrations without statistic calculation.

Dietary supplementation of Phy decreased (*P* < 0.05) fecal oocyst numbers of the *E. maxima-*infected chickens in a dose-dependent manner (**Figure 7A**). Especially, the Phy 200-fed chickens significantly reduced (*P* < 0.05) the fecal oocyst number compared to that of PC chickens from 6 to 8 dpi.

### 3.3 Experiment 3: *in vivo* studies

#### 3.3.1 Body weight and average daily gain

Before infection with *E. maxima*, BW or ADG of chickens were not changed by dietary Phy supplementation (**Table 6**). However, *E. maxima* infection without any Phy reduced (*P* < 0.05) the BW of chickens compared to that of NC. Among infection groups, chickens fed Phy at 20 mg/kg feed had a bigger BW on d 20 compared to that of the PC group. On d 22, three supplementations (20, 30, and 100 mg/kg feed) of Phy increased (*P* < 0.05) the BW of chicken compared to that of NC. Like BW, the *E. maxima* infection also decreased the ADG of chickens on d 14 to 20 compared to that of NC. Three concentrations (20, 30, and 100 mg/kg feed) of the Phy increased (*P* < 0.05) the ADG of chicken on d 14 to 20 compared to that of NC.

**Table 6:** Body weight and average daily gain chickens fed a diet supplemented with phytochemical mixture (Phy) in experiment 3.

			Phy, mg/kg feed					*P*-value		
	NC	PC	10	20	30	100	SEM	Trt	Linear	Quadratic
BW, g										
Initial	44.37	44.38	44.02	44.13	44.19	44.38	0.62	0.993	0.775	0.769
d 7	172.01	168.15	167.66	173.35	175.44	169.08	4.29	0.789	0.995	0.176
d 14	456.02	442.66	452.88	473.42	447.32	451.08	13.01	0.640	0.945	0.459
d 20 (6 dpi)	819.25^a^	669.17^c^	689.18^bc^	755.29^ab^	726.33^bc^	729.65^bc^	22.43	0.033	0.153	0.037
d 22 (8 dpi)	968.05^a^	750.08^c^	781.43^bc^	867.22^b^	836.43^b^	838.36^b^	27.24	0.018	0.098	0.016
ADG, g										
d 0 to 7	18.22	17.56	17.81	18.47	18.58	17.93	0.61	0.793	1.000	0.171
d 7 to 14	40.82	39.25	40.64	42.81	39.90	40.22	1.42	0.581	0.902	0.399
d 14 to 20	60.54^a^	37.67^c^	40.63^bc^	47.01^b^	47.42^b^	45.79^b^	2.43	0.008	0.066	0.006
d 14 to 22	64.22^a^	38.25^b^	40.91^b^	49.22^b^	48.08^b^	48.61^b^	3.62	0.027	0.088	0.054

All chickens, except for NC, were infected by oral gavage on day 14 with 1.0 × 10^4^oocysts/chicken of Eimeria maxima. SEM, the standard error of the mean; Phy, a mixture of phytochemicals used in experiment 1, NC, basal diet; PC, basal diet for E. maxima-infected chickens; Trt, treatment; BW, body weight; ADG, average daily gain; d, day; dpi, days post-infection. ^a-c^Means in the same row with different superscripts differ (P < 0.05), and the difference was re-evaluated by PDIFF option in SAS when P-value between treatments was less than 0.05.

#### 3.3.2 Jejunal cytokines, tight junction proteins, and antioxidant enzymes

**Figure 8:**
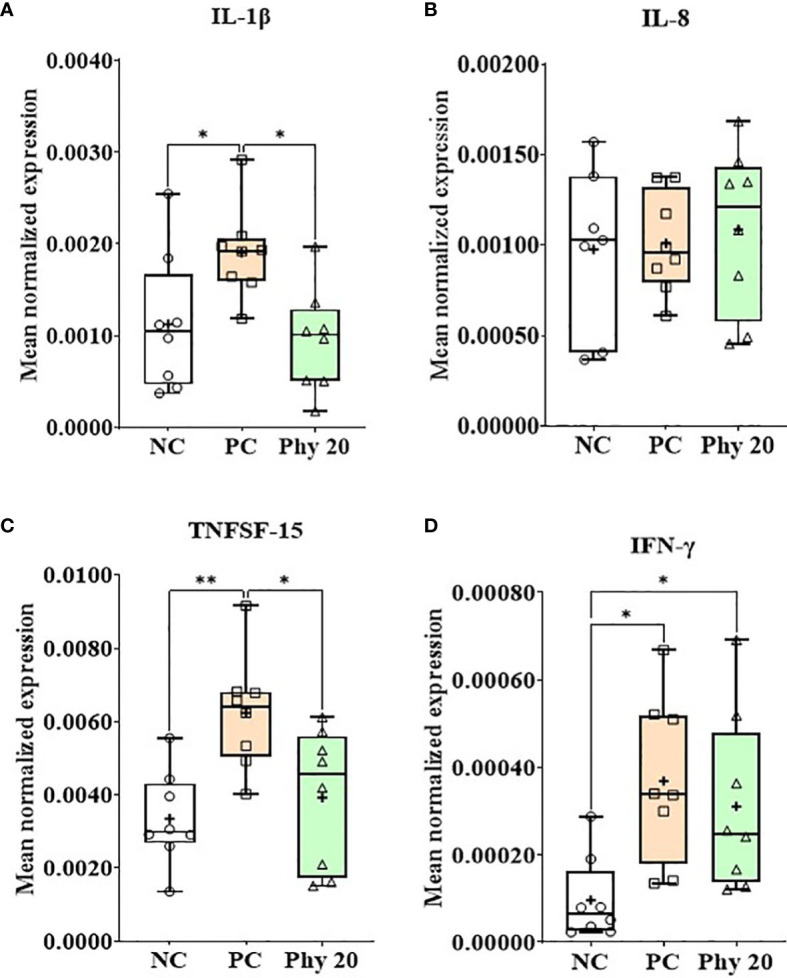
Transcripts of proinflammatory cytokines (**A**; IL-1β, **B**: IL-8, **C**: TNFSF-15 and **D**: IFN-γ) in jejunum of chickens fed diet supplemented with phytochemical during infection with *E. maxima* in experiment 3. NC, basal diet; PC, basal diet for infected chickens; Phy 20, phytochemical mixture at 20 mg/kg feed; IL, interleukin; TNFSF, tumor necrosis factor superfamily; IFN, interferon. All chickens, except for NC, were infected by oral gavage on day 14 with 1.0 × 10^4^ oocysts/chicken of *E. maxima*. *P* < 0.05 (*) and P < 0.01 (**) were considered statistically significant. The data were collected from jejunal tissues of 5 chickens per treatment on d 22 (8 days post-infection). Transcript levels of the cytokines were measured using quantitative RT-PCR and normalized to GAPDH transcript levels.

**Figure 9:**
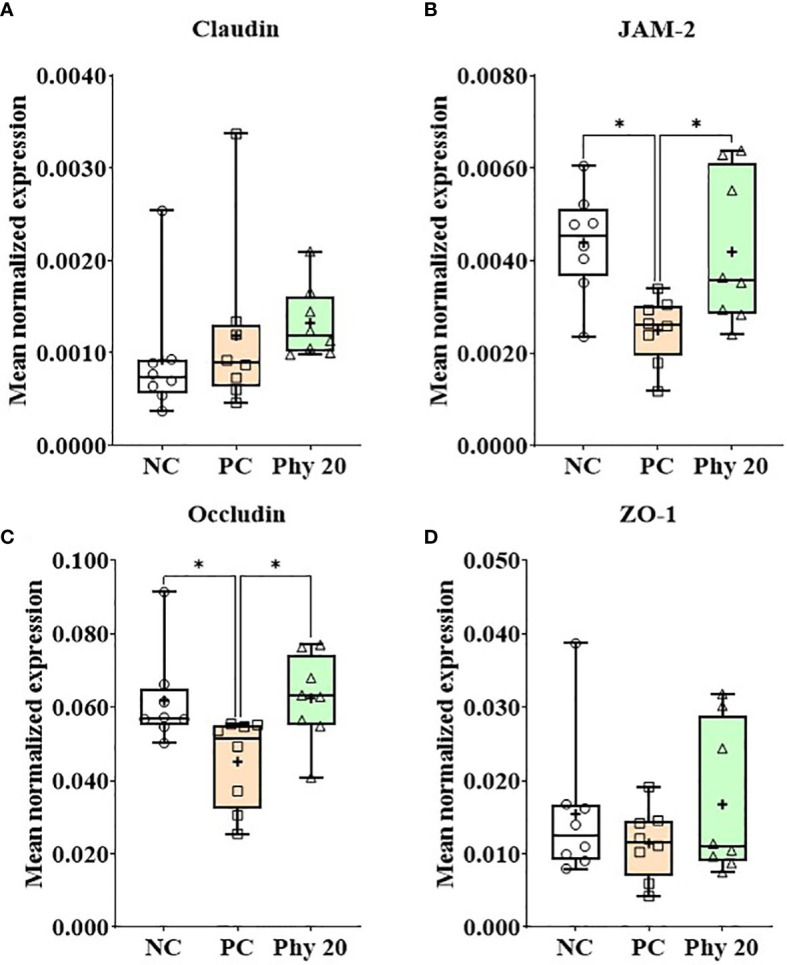
Transcripts of tight junction proteins (**A**; Claudin, **B**: JAM-2, **C**: Occludin and **D**: ZO-1) in jejunum of chickens fed diet supplemented with phytochemicals during infection with *E. maxima* in experiment 3. NC, basal diet; PC, basal diet for infected chickens; Phy 20, phytochemical mixture at 20 mg/kg feed; ZAM, junctional adhesion molecule; ZO, zonula occludins. All chickens, except for NC, were infected by oral gavage on day 14 with 1.0 × 10^4^ oocysts/chicken of *E. maxima*. *P* < 0.05 (*) was considered statistically significant. The data were collected from jejunal tissues of 5 chickens per treatment on d 22 (8 days post-infection). Transcript levels of the cytokines were measured using quantitative RT-PCR and normalized to GAPDH transcript levels.

*E. maxima* infection increased (*P* < 0.05) IL-1β, TNFSF-15, and IFN-γ expression levels in the jejunum of chickens compared to that of the NC group whereas chickens supplemented with Phy 20 had (*P* < 0.05) the lower IL-1β and TNFSF-15 levels compared to that of PC (**Figure 8**). The infection also decreased (*P* < 0.05) JAM-2 and occludin expression levels compared to that of the NC and Phy 20 recovered (*P <*0.05) these levels as much as that of the NC group (**Figure 9**). Jejunal antioxidant enzymes (**Supplementary Figure 3**) in chickens were not changed by *E. maxima* infection and dietary supplementation with Phy 20.

#### 3.3.3 Oocyst shedding and three-dimensional response surface plot

In EXP 3, chickens fed diets supplemented with Phy 10, Phy 20, and Phy 30 showed reduced oocyst numbers compared to that of PC and the decrease was dependent on the concentration of Phy (**Figure 7B**). According to the three-dimensional response surface plot that was combined with EXP 2 and 3 on the final body weight interaction between oocyst number and phytochemical supplementation, 50 mg of Phy supplementation increased the final body weight by 125% and lowered the oocyst number to less than 20% (**Figure 7C**).

## 4 Discussions

Phytochemicals promote human and animal health as demonstrated by extensive scientific literature and medical practices on traditional medicine which has focused on the disease-healing responses of the host (humans) to medicinal plants (5). Considerable studies from many laboratories including ours (5) for over 10 years have provided research data showing the beneficial effects of various phytochemicals in animals, especially in poultry. In this study, we took a novel approach to select beneficial phytochemicals that affect chicken physiological responses using *in vitro* screening system using different chicken cell lines to select phytochemicals showing desirable physiological effects on immune cells, muscle cells, and intestinal cells in chickens based on their unique biomarkers associated with cell function. In addition, we have evaluated their direct effects on parasites on select phytochemicals. Based on the effects of phytochemicals on different cell lines, we then designed novel formula that effectively mitigates coccidiosis, and special phytochemical combinations were designed to enhance host resilience during disease stress by positively affecting host physiological responses including immunity, muscle cell function, and gut health. The results of our study showed that these approaches could be successfully used to find a unique practical formulation impacting host-mediated responses involving muscle, gut, and immunity which can mitigate coccidiosis without antibiotics

Today, there are wide ranges of nutraceuticals available in the feed industry to control economically important infections in animal agriculture (3). Especially, commercial phytochemical products to reduce disease effects against coccidiosis, a major intestinal disease caused by several different species of *Eimeria* which is estimated to cause an annual economic loss of more than $14 billion worldwide (4, 12) have been reported (5). However, with an increased understanding of the mechanisms of action of many phytochemicals (3), it is becoming evident that we need to better understand the underlying mechanisms of host-pathogen interactions and the effects of various phytochemicals on host physiological responses to economically important diseases. Eventually, combinatory effects of various phytochemical feed additives that work best to reduce the harmful host response in various disease situations could effectively reduce the economic cost of animal production in the post-antibiotic era.

Phytochemicals derived from plants are natural bioactive compounds that can be incorporated into animal feed to improve productivity and are also referred to as phytobiotics or phytogenics (2). The main bioactive compounds of the phytochemicals are composed of polyphenols, and their composition and concentration vary according to the plant, parts of the plant, geographical origin, harvesting season, environmental factors, storage conditions, and processing techniques (5). The *In-vitro* model has the advantage that can predict more reliable and standardized results before performing the studies *in-vivo* (16). Notably, high concentrations of phytochemicals emerge to have cytotoxicity effects which can affect normal or healthy cells as well as cancer cells (17), therefore collecting preliminary data through an *in vitro* model may be helped to minimalize the risk to animal health in animal studies.

We have also evaluated the direct antimicrobial effects of CO, GT, and PO against *E. tenella* sporozoite and *C. perfringens* bacteria. In general, the dose ranges that showed direct cytotoxic effects against these pathogens are significantly higher than the doses mediating beneficial host physiological responses and are not practical for commercial application. The PO reduced sporozoite of *E. tenella* up to 17% at 0.1 mg/mL and 10% at 10 mg/mL. Furthermore, GO and PO showed antibacterial activity against *C. perfringens* but only at high concentrations of phytochemicals (5.0 mg/mL). Pomegranate is well known in general as having strong antioxidant and anti-inflammatory activities (18) and inhibiting apicomplexan parasite growth (19, 20) as well as gram-positive and negative bacteria growth (21). Juneja et al. (22) reported that green tea leaf extracts can reduce the potential risk of *C. perfringens* spore germination. Among active compounds in phytochemicals, polyphenols mainly play a pivotal role in antiparasitic and antibacterial activities including anthocyanidins, catechins [epicatechin (EC), epicatechin-3-gallate (ECG), epigallocatechin (EGC), gallocatechin, epigallocatechin-3-gallate (EGCG), catechin gallate], cinnamic aldehyde, gallic acid, tannins (gallotannins and ellagitannins), and theaflavins [theaflavin (TF), TF-3-O-gallate(TF3G), TF-3′-O-gallate (TF3′G), TF-3,3′-O-digallate (TFdG)] (23–28). CO did not show any antimicrobial activities even though they are known as having similar bioactive constituents (polyphenols) as green tea extract and pomegranate. This discrepancy may be contributed by the variety of botanic components, as well as the variability of the animal conditions, the presence of a pathogen, and methods of producing phytochemicals (6). LPS treatment of chicken epithelial cells dramatically increased proinflammatory cytokines such as IL-1β and IL-8 (29, 30). The IL-1β mediates the inflammatory responses for a wide range and the IL-8 is responsible for recruiting neutrophils (heterophils) to the inflamed site (31). Administration of CO and GT suppress LPS-induced IL-1β in chicken epithelial cells and PO administration reduced LPS-induced IL-8. Plant-derived phytochemicals showed anti-inflammatory effects altering the cytokine levels in the intestine in chicken and pigs following treatment with various pathogens such as LPS (32), *Eimeria* spp (33), *Salmonella* spp (31), and *E. coli* (34).

The optimum regulation of intestinal permeability through intricate mechanisms of physiological and immunologic events on the intestinal epithelial barrier is an inevitable requirement to allow efficient digestion, absorption, and passage of nutrients as well as restrict the entry of larger molecules, such as antigens (35). The paracellular pathway for intestinal permeability is manipulated by TJs, adherens junctions, and desmosomes (36, 37). Among them, TJs are composed of multiprotein complexes such as membrane proteins (claudin and occludin), and scaffold proteins (ZO-I and II) (38). In the current study, GT improved occludin expression levels in IEC by 1.97-fold. Park et al. (28) and Lagha and Grenier (39), reported that green tea extract improved occludin levels similarly to our observation by around 2-fold. They also revealed that these beneficial results were due to EGCG and TF3′G from green tea extract. Sundstrom et al. (40) and Tash et al. (41) demonstrated that the altered occludin level regulates TJ barriers in response to phosphorylation, cytokines, and growth factors. However, the ZO-1 level was not changed by these phytochemical administrations. Unlike our result, ZO-1 is generally known as a TJ protein that mediated interactions with occludin and F-actin. Further studies will be necessary to better understand the molecular mechanisms involved in the phytochemical regulation of TJ proteins.

In the current study, the gene expression levels of MyoG and Pax7 were measured to investigate how phytochemicals affect muscle growth related to the growth of chickens. Lower FBS concentration [0.5% FBS in QMC (13) and 2% FBS in PMC (15)] to induce myogenic differentiation improved MyoG expression levels in QMC and PMC by more than 5-fold and 2-fold, respectively. In this study, only PO at a 10 mg dose showed that MyoG in QMC was improved by around 45% compared to the non-treated group when 0.5% FBS was used. Unlike our expectation, Pax7 was also increased in the groups that induced cell differentiation by lowering the FBS concentration. According to Rudnicki et al. (42), satellite cells are precursor or stem cells of skeletal muscles as mononuclear cells, divided into four stages (quiescence, proliferation, renewal self, and differentiation) of growth. Every stage expresses its myogenic regulatory factors (Pax7, MyoD, Myf5, myogenin, and MRF4). When muscle production occurs, satellite cells, which were normally quiescence (Pax7^+^MyoD^-^), enter the cell cycle again, increasing the expression of MyoD and Myf5 (Pax7^+^MyoD^+^). For the differentiation of these satellite cells, Pax7 is reduced and myogenin (Pax7^-^MyoD^+^Myogenin^+^) is increased to produce a new nuclear or myotube (43). Therefore, the current result of QMC indicates preferential cell differentiation because the MyoG level was more than 5-fold compared to that (more than 2-fold) of Pax7. PMC cells were in a cell proliferation phase based on the ratio of Pax to MyoG which was almost 1:1. To induce differentiation of PMC, it may be possible to extend incubation time or lower FBS concentration further. Although most of the data came from studies in mice, polyphenols showed a positive effect on muscle development (44).

In this study (EXP 1), each phytochemical showed beneficial effects on host inflammatory response, TJ proteins, and/or muscle growth factors. Therefore, a mixture of CO, GT, and PO provides beneficial effects against coccidiosis in poultry. The effects of selected phytochemical mixture were evaluated for its efficacy on growth performance and intestinal health of young broiler chickens infected with *E. maxima* (EXP 2 and 3) in commercial broiler chickens. In previous studies, dietary supplementation of young broiler chickens with a single phytochemical has shown a beneficial effect on growth, intestinal immune responses, and intestinal epithelial integrity of chicken infected with *Eimeria* spp (5, 45–47). However, a single phytochemical feed additive sometimes failed to exert beneficial effects on host, and in some cases, its effects were harmful (48–51). Richards et al. (52) and Rizeq et al. (53) suggested that a combination of phytochemical feed additives may promote a synergy effect. As shown in our study (EXP 2), BWs on d 14 (before infection) of chickens fed dietary Phy 50 and 100 were improved by 7% and 6%, respectively, compared to that of chickens fed basal diet (NC and PC). However, chickens fed dietary Phy 200 showed decreased BW by 4.2% than that of NC and PC. Phytochemicals may have a biphasic dose-response relationship, showing a beneficial effect at lower doses but may have adverse effects at higher doses. This could be due to a variety of reasons, such as saturation of absorption, metabolism, or elimination pathways, as well as the. activation of different cellular mechanisms that can lead to toxicity or cellular damage (5). Infection (PC) with *E. maxima* dramatically reduced the BW at 6 dpi by 22% compared to that of NC while Phy 100 enhanced the BW of PC by 13%. The effect of Phy supplementation was long-lasting since Phy-treated chickens showed a greater BW at 8 dpi by 7.5% in Phy 50 and 15% in Phy 100 when compared to that of PC.

In EXP 3, the effect of the phytochemical supplementation was evaluated in the lower concentration of Phy (10 to 100 mg/kg feed) than in EXP 2. All phytochemical-supplemented groups did not show a negative effect of BW on d 7 and 14 (before infection) compared to that of NC and PC. However, unlike our expectation, Phy 100 did not have any difference compared to that of NC or PC. *E. maxima* infection in EXP 3 also reduced the BW at 6 dpi by 22% like EXP 2 and Phy 20 improved the BW by 13% compared to that of the PC group. Especially, in BW at 8 dpi, all Phy groups except 10 mg improved the BW of chickens by 16% in 20 mg, 12% in 30 mg, and 12% in 100 mg when compared to that of the PC group. There was no mortality by *Eimeria* infection during both EXP 2 and 3. In this study, we chose the infecting dose of *Eimeria* to induce a mild infection to avoid severe coccidiosis and to show the beneficial effects of phytonutrients. In this study, the infection decreased growth by approximately 22%, which we believe to be an appropriate level of infection based on our extensive previous experience (1, 13–15).

Distal jejunal samples were collected at 8 dpi (EXP 2 and 3) to analyze proinflammatory cytokines, TJ proteins, and antioxidant enzymes. Among them, samples of Phy 100 in EXP 2 and Phy 20 in EXP 3 were selected for these analyses because each group showed the best growth in each EXP. Infection with *E. maxima* induced all cytokines except IL-8 in both EXPs at 8 dpi. Both Phy 100 and Phy 20 treatment groups showed mitigated *E. maxima*-induced inflammatory responses by suppressing proinflammatory cytokines (IL-1β and TNFSF-15) in the jejunum of chickens. In EXP 2, Phy 100 group did not show any changes in TJ protein expression after *E maxima* infection, although the infection with *E. maxima* increased intestinal permeability by decreasing the levels of JAM-2 and occludin and then the Phy 20 recovered these proteins in EXP 3.

In the case of antioxidant enzymes, no phytochemical-supplemented groups show any beneficial effect in both studies. When measuring host immune responses and barrier integrity in the intestine to evaluate the growth-promoting effects of feed additives in *Eimeria* infections, it is very important to choose the optimum time points for sample collection since host-parasite interaction changes with the life cycle of *Eimeria*. In chickens, infection with *Eimeria* spp reaches the peak response between 4 and 6 dpi even though the magnitude of infection will be determined by the infecting dose and genetics of the host (54). In this study, the sample collection was done at 8 dpi based on our established animal protocol. Fecal oocyst numbers gradually decreased as the concentrations of Phy increased although Phy 200 showed a negative effect on BW in EXP 2. In EXP 3, a lower concentration (10 to 30 mg/kg feed) of Phy reduced the oocyst number in fecal samples than that of PC.

In the current study, high concentrations of phytochemicals may have exerted toxic effects against parasites and bacteria as we have seen in the *in vitro* antimicrobial assays, but it is not clear what effects these high-dose treatments had on host response in animal trials, especially with respect to body weight and parasite fecundity after coccidiosis challenge. Thus, it is important to consider the effect of phytochemical doses on their physiological (nutritional) effects to maintain optimal health. In the three-dimensional plot, the average values of PC on final body weight and oocyst number were considered 100%. The surface was completed with each value of BW, oocyst number, and Phy concentration without statistic calculation, thus the figure shows a simple tendency. Therefore, Phy at around 50 mg increased BW by around more than 20% and reduced the oocyst number by less than 20%.

In conclusion, this is the first study demonstrating the development of an effective phytochemical feed additive formula based on the *in vitro* evaluation of different physiological host functions using chicken cell lines to mitigate coccidiosis. This paper showed that the combination of CO, GT, and PO exerted beneficial effects on mitigating coccidiosis response by impacting host immunity, muscle growth, and gut integrity. Although these phytochemicals showed antiparasitic and antibacterial activities against *E. tenella* and *C. perfringens*, respectively, at higher phytochemical doses, their beneficial effects in coccidiosis trials were due to phytochemical-induced mitigation of host disease responses including reduced inflammation, enhance gut function and facilitated muscle growth. Their combinatory effects on promoting gut health significantly reduced parasite-induced gut injury after coccidiosis infection. These findings will provide a scientific rationale to develop a science-based antibiotic-independent strategy to mitigate coccidiosis response in poultry production to reduce the economic cost associated with commercial broiler production.

## Data availability statement

The original contributions presented in the study are included in the article/**Supplementary Material**. Further inquiries can be directed to the corresponding author.

## Ethics statement

The animal study was reviewed and approved by The Beltsville Agricultural Research Center Institutional Animal Care and Use Committee.

## Author contributions

IP and HL designed the research. HL supervised the research. IP, and HN conducted the *in vitro* research. IP, YL, and SW conducted the *in vivo* research. IP analyzed data and drafted the manuscript. HL supervised the research and edited the manuscript. IP, HN, SW, YL, EW, SR, and HL had responsibility for the content. All authors contributed to the article and approved the submitted version.
