# Supplementary material for: Host-mediated beneficial effects of phytochemicals for prevention of avian coccidiosis
Source: Front Immunol. 2023 Jun 2;14:1145367. doi: 10.3389/fimmu.2023.1145367 (PMC10272459; doi:10.3389/fimmu.2023.1145367)
Supplement: Supplementary file 1 [file DataSheet_1.docx]

Supplementary Material

Host-mediated beneficial effects of phytochemicals for prevention of avian coccidiosis

Inkyung Park, Hyoyoun Nam, Samiru S. Wickramasuria, Youngsub Lee, Emma H. Wall, Sripathy Ravichandran, and Hyun S. Lillehoj

*** Correspondence:** Dr. Hyun S. Lillehoj: hyun.lillehoj@usda.gov

# Supplementary Figures and Tables

## Supplementary Figures


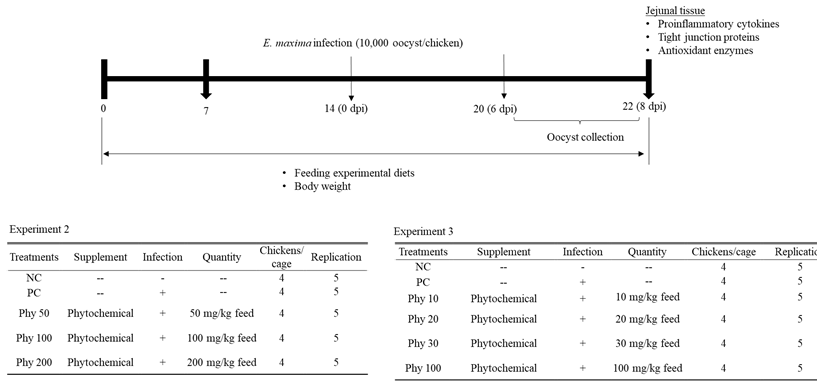


**Supplementary Figure 1.** Schematic outline of the experimental design in experiment 2 and 3.


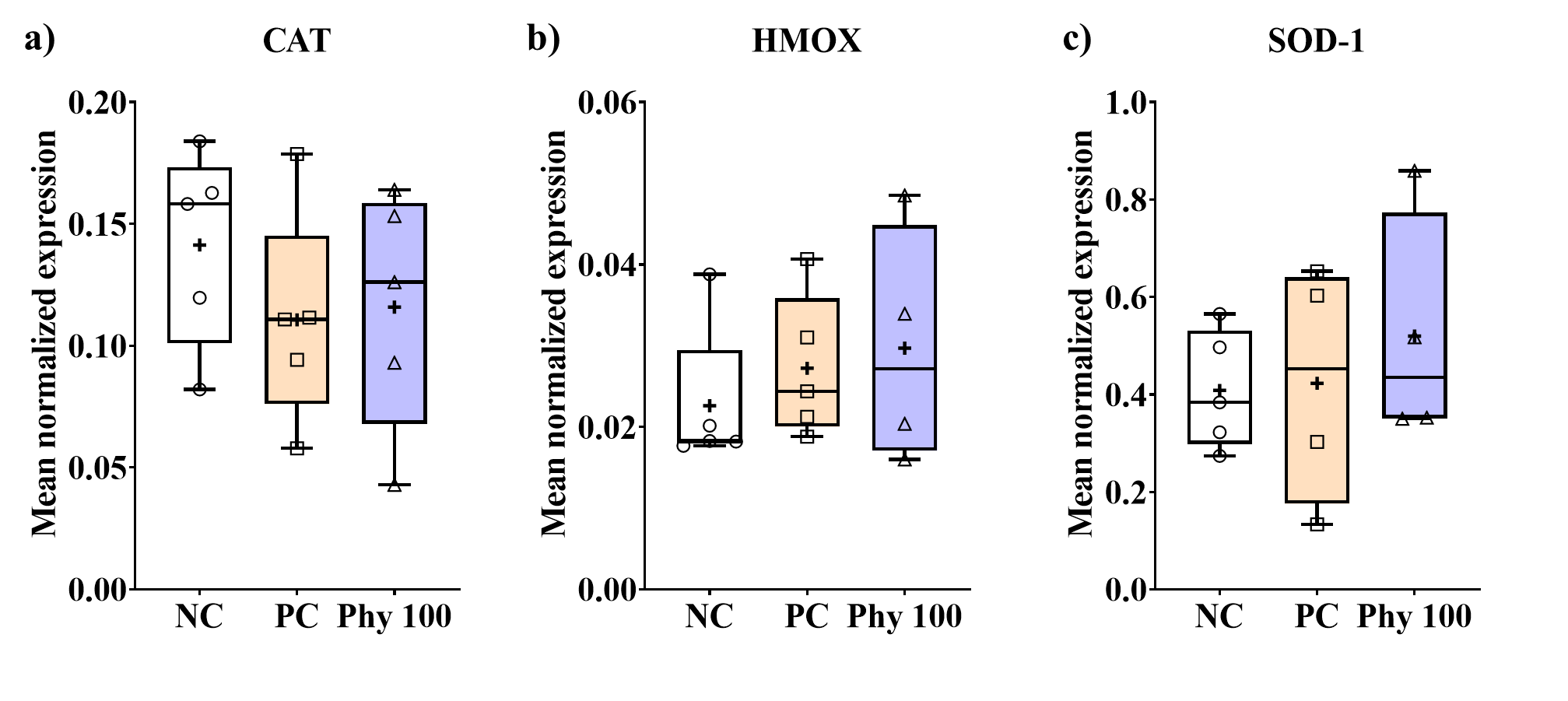


**Supplementary Figure 2.** Transcripts of antioxidant enzymes in jejunum of chickens fed diet supplemented with phytochemical during infection with *Eimeria maxima* in experiment 2. NC, basal diet; PC, basal diet for infected chickens; Phy 100, phytochemical mixture at 100 mg/kg feed; CAT, catalase; HMOX, heme oxygenase; SOD, superoxide dismutase. All chickens, except for NC, were infected by oral gavage on day 14 with 1.0 × 10^4^ oocysts/chicken of *E. maxima*. The data were collected from jejunal tissues of 5 chickens per treatment on d 22 (8 days post-infection). Transcript levels of the cytokines were measured using quantitative RT-PCR and normalized to GAPDH transcript levels.


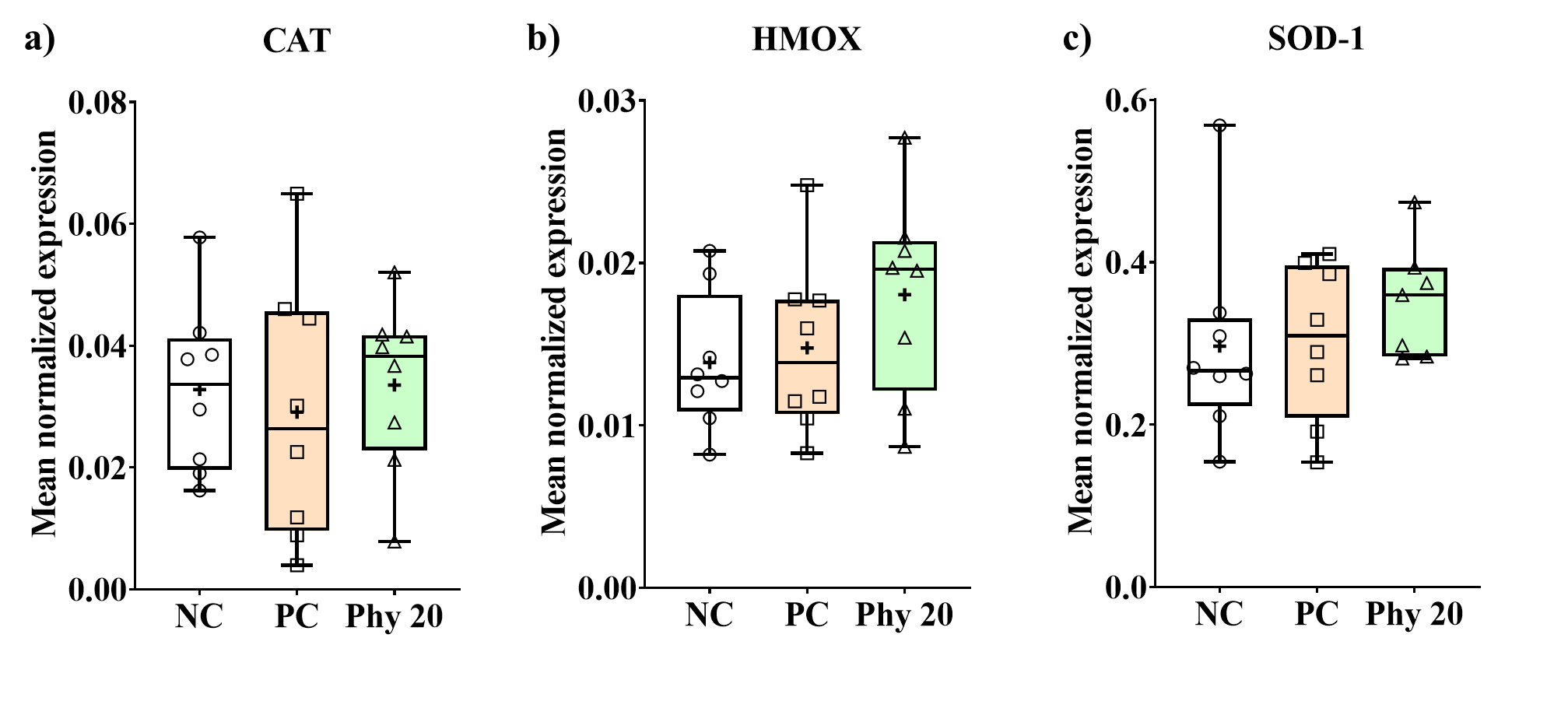


**Supplementary Figure 3.** Transcripts of antioxidant enzymes in jejunum of chickens fed diet supplemented with phytochemical during infection with *Eimeria maxima* in experiment 3. NC, basal diet; PC, basal diet for infected chickens; Phy 20, phytochemical mixture at 20 mg/kg feed; CAT, catalase; HMOX, heme oxygenase; SOD, superoxide dismutase. All chickens, except for NC, were infected by oral gavage on day 14 with 1.0 × 10^4^ oocysts/chicken of *E. maxima*. The data were collected from jejunal tissues of 5 chickens per treatment on d 22 (8 days post-infection). Transcript levels of the cytokines were measured using quantitative RT-PCR and normalized to GAPDH transcript levels.

## 1.2. Supplementary Table

**Supplementary Table** 1. Ingredient composition of basal diet (as-fed basis, %, unless otherwise indicated)

| Ingredients (%) | Basal diet |
| --- | --- |
| Corn | 55.78 |
| Soybean meal | 37.03 |
| Soybean oil | 2.97 |
| Dicalcium phosphate | 1.80 |
| Calcium carbonate | 1.51 |
| Salt | 0.38 |
| Poultry Vit Mix^1^ | 0.22 |
| Poultry Mineral Mix^2^ | 0.15 |
| DL-Methionine | 0.10 |
| Choline-chloride, 60% | 0.06 |
| Total | 100.00 |
| Calculated values (%) |  |
| CP, % | 24.00 |
| Ca, % | 1.20 |
| AP, % | 0.51 |
| Lys, % | 1.40 |
| Met, % | 0.49 |
| Cys + Met, % | 0.80 |
| ME, Mcal/kg | 3.5 |

^1^ Vitamin mixture provided the following nutrients per kg of diet: vitamin A, 2,000 IU; vitamin D3, 22 IU; vitamin E, 16 mg; vitamin K, 0.1 mg; vitamin B1, 3.4 mg; vitamin B2, 1.8 mg; vitamin B6, 6.4 mg; vitamin B12, 0.013 mg; biotin, 0.17 mg; pantothenic acid, 8.7 mg; folic acid, 0.8 mg; niacin, 23.8 mg.

^2^ Mineral mixture provided the following nutrients per kg of diet: Fe, 400 mg; Zn, 220 mg; Mn, 180 mg; Co, 1.3 mg; Cu, 21 mg; Se, 0.2 mg. CP = crude protein, AP = available phosphorus.
